# New CD1d agonists: Synthesis and biological activity of 6″-triazole-substituted α-galactosyl ceramides

**DOI:** 10.1016/j.bmcl.2012.05.009

**Published:** 2012-07-01

**Authors:** Peter J. Jervis, Lisa M. Graham, Erin L. Foster, Liam R. Cox, Steven A. Porcelli, Gurdyal S. Besra

**Affiliations:** a School of Biosciences, University of Birmingham, Edgbaston, Birmingham B15 2TT, UK; b School of Chemistry, University of Birmingham, Edgbaston, Birmingham B15 2TT, UK; c Department of Microbiology and Immunology, Albert Einstein College of Medicine, Yeshiva University, Bronx, NY 10461, USA

**Keywords:** α-GalCer, CD1d, *i*NKT cell, Triazole, Click chemistry

## Abstract

Huisgen [3+2] dipolar cycloaddition of 6″-azido-6**″**-deoxy-α-galactosyl ceramide **11** with a range of alkynes (or a benzyne precursor) yielded a series of triazole-containing α-galactosyl ceramide (α-GalCer) analogues in high yield. These α-GalCer analogues and the precursor azide **11** were tested for their ability to activate *i*NKT cells and stimulate IL-2 cytokine secretion in vitro, and IFN-γ and IL-4 cytokine secretion in vivo. Some of these analogues, specifically **11**, **12b**, **12f** and **13**, were more potent IL-2 stimulators than the prototypical CD1d agonist, α-GalCer **1**. In terms of any cytokine bias, most of the triazole-containing analogues exhibited a small Th2 cytokine-biasing response relative to that shown by α-GalCer **1**. In contrast, the cycloaddition precursor, namely azide **11**, provided a small Th1 cytokine-biasing response.

## Graphical abstract

6″-Triazole-substituted-*α*-galactosyl ceramides **12a**–**f**, **13**, **14** and **15** have been synthesised. Along with azide precursor **11**, their ability to activate *i*NKT cells and stimulate the production of IL-2 cytokines in vitro, and IFN-γ and IL-4 cytokines in vivo, has been evaluated.

α-GalCer (KRN7000, **1**, Fig. 1) is a simplified synthetic analogue of the naturally occurring agelasphins (including AGL-9b, **2**, Fig. 1), which were isolated from the marine sponge *Agelas mauritianus*.^1^ α-GalCer has become the prototypical ligand for studying the CD1d-restricted activation of invariant Natural Killer T cells (*i*NKT cells).^2^ CD1d is an MHC-like protein located on the surface of various antigen-presenting cells, including dendritic cells and macrophages.^3^ α-GalCer (**1**) binds through its two long lipid chains into two deep hydrophobic pockets of the CD1d molecule, to form an α-GalCer–CD1d complex.^4^ CD1d then presents this glycolipid to T-cell receptors (TCRs) located on *i*NKT cells, an event which elicits an immune response through the release of both pro-inflammatory (Th1 (IFN-γ)) and regulatory (Th2 (IL-4)) cytokines.^5,6^ The release of Th1 cytokines is associated with antitumour and antimicrobial functions,^7^ whilst the release of Th2 cytokines is implicated in alleviation of autoimmune diseases^8–10^ such as multiple sclerosis^11^ and arthritis.^12^ When both Th1 and Th2 cytokines are released together, however, their effects are counteractive, providing unpredictable biological effects.^13^ The absence of a Th1/Th2 cytokine bias has hindered the therapeutic application of α-GalCer and encouraged the search for analogues of this CD1d agonist, which induce a more biased Th1/Th2 response.^14^ Most modifications to α-GalCer have been to the ceramide portion of the molecule and some important examples are shown in Figure 1. For example, truncation of either the fatty acid chain (**3**)^15^ or the sphingosine chain (OCH, **4**)^16^ produces a Th2 cytokine-biasing response. A Th2 cytokine-biasing response is also observed when unsaturation is incorporated into the acyl chain (α-GalCer C20:2, **5**).^17^ Switching the anomeric oxygen atom for a methylene group (α-*C*-GalCer, **6**) provides an example of a Th1-skewing analogue.^18^ Despite the efforts of many laboratories, the factors which determine the nature and extent of any cytokine bias remain only partially understood.^19^

The design of many glycolipid analogues has been guided by the published crystal structures of the CD1d–KRN7000 complex and the TCR–KRN7000–CD1d complex.^20,21^ These crystal structures reveal the hydroxyl group at the 6-position of the sugar head-group is the only hydroxyl group that is not directly involved in hydrogen bonding to the CD1d protein or the TCR of the *i*NKT cell. As such, α-galactosyl ceramides in which the hydroxyl group at the 6-position of the sugar head group has been modified, have become attractive synthetic targets. Indeed, many groups have shown that the TCR–glycolipid–CD1d interaction is tolerant to derivatisation at this position of the molecule.^22,23^ For example, Tashiro et al. found that methyl ether **7** (RCAI-61) skews the cytokine profile towards a Th1 response.22a The activity of **7** has been rationalised by the methylation disrupting a hydrogen bond between the oxygen atom of the 4″-hydroxyl group and the hydrogen atom of the 6″-hydroxyl group, thus rendering the 4″-hydroxyl group more available for TCR recognition.22a

The incorporation of nitrogen-containing functionalities into the 6-position of the sugar has also proven to be worthwhile.^23^ From a practical point of view, incorporating a nitrogen functionality into this position of the glycolipid serves to increase substrate solubility (α-GalCer is very poorly soluble in most organic solvents and water), facilitating easier synthesis, purification, handling and biological administration.23a More importantly, this type of structural change has also been shown to produce desirable biological effects. For example, Trappeniers et al. have shown that analogues containing aryl amides and ureas at the 6″-position (e.g., **8** and **9**) can skew the cytokine profile in favour of a Th1 cytokine response.23b A crystal structure of the CD1d–**9**–TCR complex reveals the urea substituent residing in a hydrophobic pocket, effectively providing an extra site for binding. In addition, the naphthyl ring of **9** is likely to be involved in π–π stacking with the electron-rich indole ring of the proximal Trp153.23c Interestingly, when the 6-amido group is linked to a poly(ethylene glycol) chain, as in amide analogue **10**, the cytokine profile is reversed such that a Th2-biasing cytokine response is now observed (Fig. 2).23c

We have recently developed a synthesis of a 6″-azido-6″-deoxy-α-galactosyl ceramide (**11**), which is a useful precursor for the synthesis of 6″-*N*-derivatised α-GalCer analogues (Fig. 3).^24^ Reducing the azide functionality provides the corresponding amine, potentially allowing ready access to amides, sulfonamides, ureas, thioureas and secondary amines. Azide **11** is also primed for more direct modification utilising click chemistry.^25–27^ Alkyne–azide [3+2] dipolar cycloaddition reactions are highly chemoselective, and we predicted that azide **11** should react readily without the need to protect the hydroxyl groups, providing ready access to a library of 1,2,3-triazole-containing KRN7000 derivatives from the large range of inexpensive, terminal alkynes that are available.

1,2,3-Triazoles are considered to be non-hydrolysable bioisosteres of the amide bond.28a,29 In terms of atom positioning, the 1,4-disubstituted 1,2,3-triazole mimics the s*-cis* amide rotamer, whilst the 1,5-disubstituted analogue mimics the s-*trans* amide rotamer.28b The similarities and differences in hydrogen bonding of these isosteres have been described recently by Tron et al.28b Both 1,4- and 1,5-disubstituted triazoles can be accessed by judicious choice of reagents and reaction conditions.^25–27^

As part of a wider programme directed towards generating CD1d agonists,^30^ and to test the scope of azide **11** as a cycloaddition partner, we embarked on the preparation of a range of 6″-triazole-substituted α-GalCer analogues. In the first instance, we focused on the 1,4-disubstitution pattern of the triazole, which was accessed via copper-catalysed click chemistry.^25,26^ Heating equimolar quantities of azide **11** with different acetylenes of varying steric demand and hydrophilicity, in the presence of CuSO_4_ and sodium ascorbate, provided the desired 1,4-disubstituted triazoles **12a**–**f** in excellent yields. In order to assess the effect of regiochemistry on the biological activity, we were also keen to access the alternative regioisomer of triazole **12a**.^27^ To this end, heating azide **11** with phenyl acetylene in the presence of 5 mol % Cp_∗_Ru(PPh_3_)_2_Cl, provided 1,5-disubstituted triazole **13** in 78% yield. This ruthenium catalyst is also reported to mediate the reaction with internal alkynes and indeed, employing diphenyl acetylene yielded the 1,4,5-trisubstituted triazole **14** in 72% yield.^27^ Finally, benzotriazole-containing analogue **15** was also prepared in 69% yield from an in situ-generated benzyne intermediate, by treating azide **11** with 2-(trimethylsilyl)phenyl trifluoromethanesulfonate in the presence of TBAF (Scheme 1).^31^

α-GalCer analogues **11**, **12a**–**f** and **13**–**15** were initially tested in vitro for their ability to stimulate IL-2 production by murine *i*NKT hybridoma cells (Fig. 4).^19^ Analogues **11**, **12b**, **12f** and **13** were all more active than α-GalCer **1**, whilst the remaining analogues tested displayed significantly reduced activity relative to α-GalCer. There were interesting differences in activity between the benzene ring-containing analogues, in that triazole **13**, which contains the 1,5-disubstitution pattern on the triazole ring, was far more active than 1,4-disubstituted triazole **12a**, 1,4,5-trisubstituted triazole **14** and benzotriazole **15**. There is also a striking difference in activity between the alkyl-substituted triazoles, with triazole **12b** being far more active than triazole **12c**, despite only a four-carbon difference in alkyl chain length between these two molecules.

α-GalCer analogues **11**, **12a**–**f**, **13**–**15** were next tested for their ability to stimulate cytokine production in vivo by measuring the serum IL-4 and IFN-γ levels, at 2 h and 24 h, respectively, in C57BL/6 mice following intraperitoneal injection (Fig. 5).^33^ In terms of overall cytokine release, all the analogues tested were active in these in vivo experiments, with triazole **13** (containing the 1,5-disubstitution pattern) providing the highest levels of IL-4 secretion, and azide **11** providing the highest level of IFN-γ secretion. Again, there are interesting differences in the amount of cytokine production elicited by the two alkyl-substituted analogues (**12b** and **12c**). Triazole **12b** (containing an octyl chain) provides more than double the amount of both IL-4 and IFN-γ cytokines than does triazole **12c** (containing a dodecyl chain). That a relatively small change to the length of the alkyl chain attached to the triazole unit is having a large effect on the levels of cytokine production, might be indicative of the amount of space available within an extra hydrophobic binding site. Triazole **12f** (containing a PEG-8 substituent), which contains a much longer (but hydrophilic) chain length, elicits higher levels of cytokine production than both **12b** and **12c**, providing similar results to α-GalCer in both the IL-4 and IFN-γ assays. The ethylene glycol chain might not be expected to benefit from the presence of an extra hydrophobic binding site, but might instead exert its effects on activity through increased solubility.

In order to analyse the cytokine bias of our α-GalCer analogues, the ratio of IFN-γ to IL-4 secretion was calculated and compared with that for α-GalCer **1** (Fig. 6). Interestingly, analogues **12a** and **13**, which might be considered bioisosteric to the Th1-skewing amide **8** and urea **9**, were found instead to possess a small Th2 cytokine-biasing response, relative to α-GalCer. In fact, all of the triazole-containing analogues showed either a small Th2 cytokine-biasing response (being most pronounced in the case of benzotriazole **15**) or were similar in their behaviour to α-GalCer. The only Th1-biasing analogue tested was the synthetic precursor of our triazole analogues, namely azide **11**. Triazole **12f**, which might have been expected to be a Th2 cytokine-biasing analogue owing to its structural similarities with the Th2 cytokine-biasing amide **10**, exhibited no significant cytokine bias relative to α-GalCer.

In summary, click reactions of azide **11** with various alkynes provided a library of 6″-triazole-substituted α-GalCer analogues.^34^ The ability of these analogues to stimulate the production of IL-2 varied significantly with small changes to the nature of the triazole substituent. All of these new triazole-containing α-GalCer analogues stimulated both IL-4 and IFN-γ secretion in vivo, and elicited either a small Th2 cytokine-biasing response or were similar in their cytokine profile to α-GalCer. The click chemistry precursor, azide **11** was also tested, and found to provide a small Th1 cytokine-biasing response. These initial biological results suggest that subtle structural changes to this part of α-GalCer can have a significant effect on the observed cytokine bias. Future work will involve further SAR studies and the use of computer modelling in order to better understand how these molecules interact with the CD1d molecule and the *i*NKT-cell TCR.

## Figures and Tables

**Figure 1:**
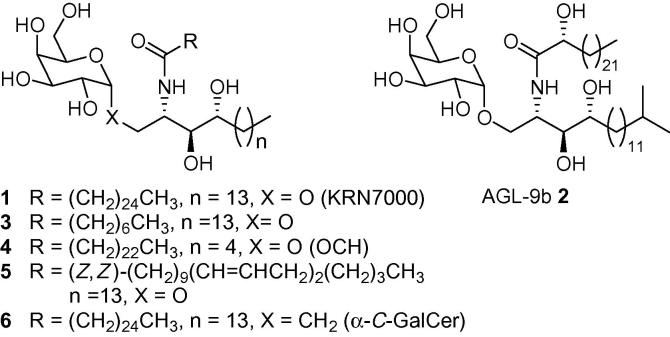
Prototypical KRN7000 (**1**), naturally occurring AGL-9b (**2**) and biologically active analogues **3**–**6**.

**Figure 2:**
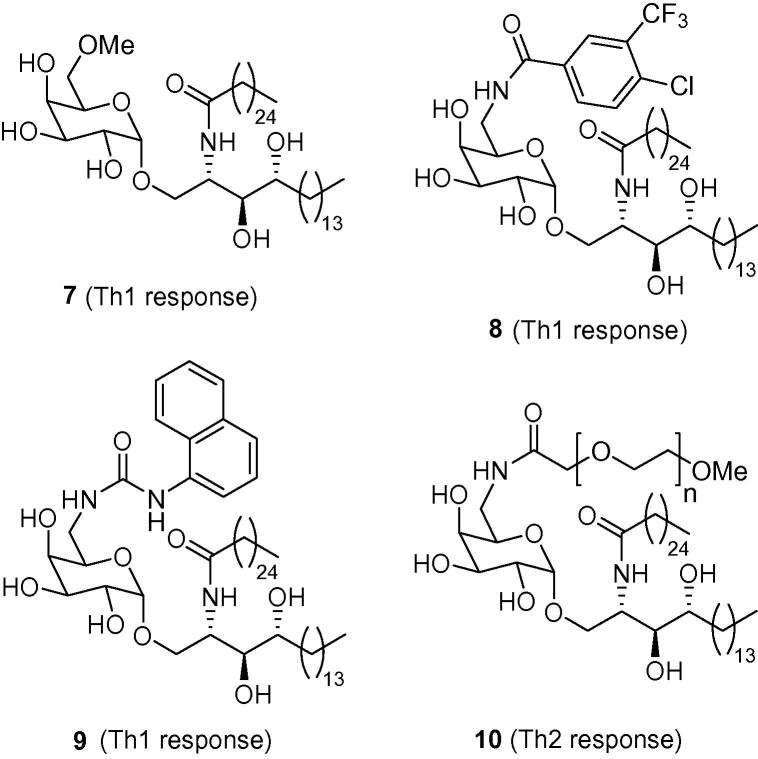
Biologically active 6″-derivatised α-galactosyl ceramide analogues **7**–**10**.

**Figure 3:**
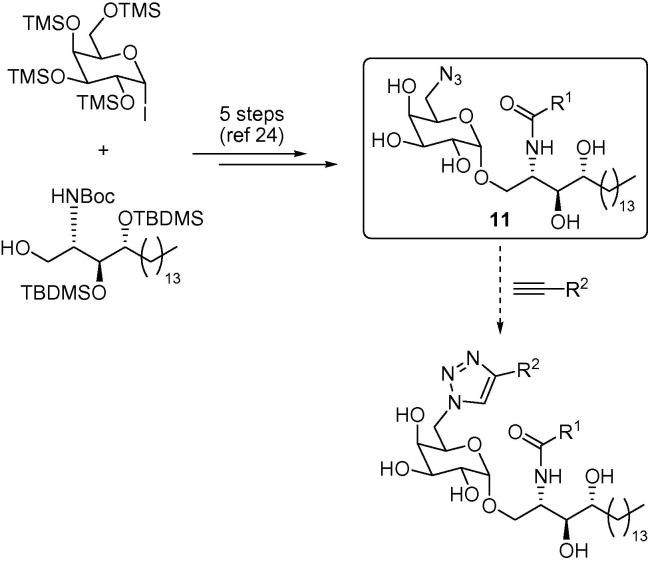
A route to 6-*N*-derivatised α-GalCer analogues, which allows late-stage modification of both the 6-*N*-substituent and the fatty acid group of the ceramide.

**Figure 4:**
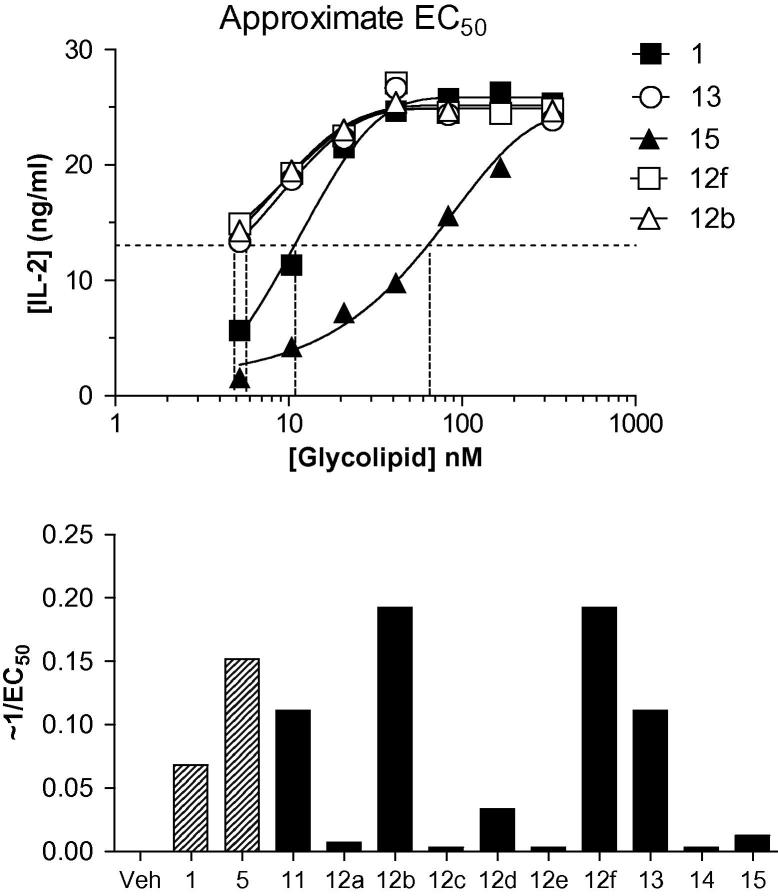
EC_50_ measurements using mouse *i*NKT hybridoma DN3A4–1.2. Top panel: Glycolipids were titrated over ∼3 log range and IL-2 levels at 24 h were measured in culture supernatant by ELISA. After nonlinear curve fitting, the glycolipid concentration giving 50% maximum *y*-values was estimated. The horizontal dotted line is the approximate 50% maximum response for the assay, and the vertical dashed lines show the extrapolation to the *x*-axis values for determination of EC_50_. Representative examples are shown for three glycolipid antigens, including the positive control KRN7000 (**1**). Bottom panel: Relative potencies for all compounds, based on 1/EC_50_ values as determined using mouse *i*NKT hybridoma DN3A4–1.2 and the method for measuring of EC_50_ values as described above.^19^

**Figure 5:**
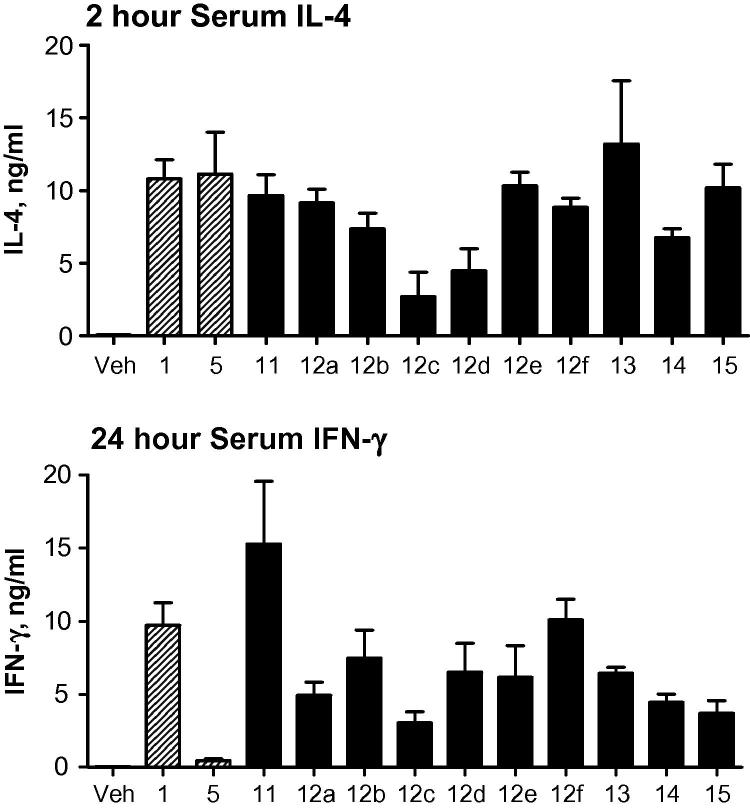
IL-4 (top panel) and IFN-γ (bottom panel) secretion after intraperitoneal injection of α-GalCer **1**, α-GalCer (C20:2) **5**, **11**, **12a**–**f** and **13**–**15** in mice.^33^

**Figure 6:**
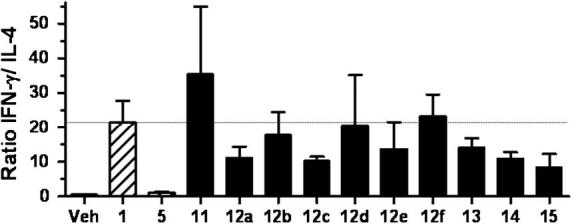
IFN-γ:IL-4 ratios for α-GalCer **1**, α-GalCer (C20:2) **5**, **11**, **12a**–**f** and **13**–**15** in mice. Normalised so that the value for α-GalCer (C20:2) **5** is 1.

**Scheme 1:**
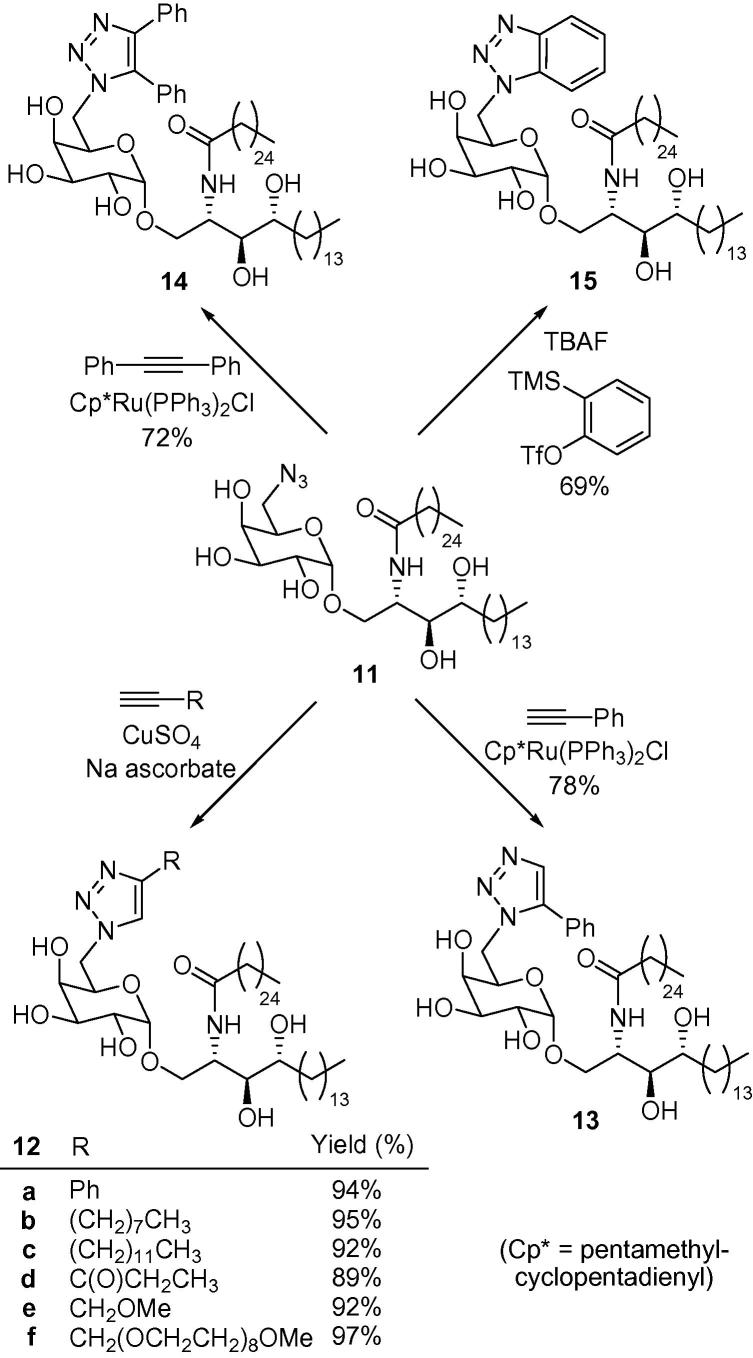
Synthesis of triazole-containing α-GalCer analogues.^32^

## Supplementary data

Supplementary data General procedure and spectral data.
