# Supplementary material for: New CD1d agonists: Synthesis and biological activity of 6″-triazole-substituted α-galactosyl ceramides
Source: Bioorg Med Chem Lett. 2012 Jul 1;22(13):4348–52. doi: 10.1016/j.bmcl.2012.05.009 (PMC3401990; doi:10.1016/j.bmcl.2012.05.009)
Supplement: Supplementary data — General procedure and spectral data. [file mmc1.doc]

New CD1d Agonists: Synthesis and Biological Activity of 6”-Triazole-Substituted α-Galactosyl Ceramides

Peter J. Jervis^a,b^, Lisa M. Graham^c^, Erin L. Foster^c^, Liam R. Cox^b^, Steven A. Porcelli^c^, Gurdyal S. Besra^a^*

*^a^School of Biosciences, University of Birmingham, Edgbaston, Birmingham B15 2TT, U.K., ^b^School of Chemistry, University of Birmingham, Edgbaston, Birmingham B15 2TT, U.K., ^c^Department of Microbiology and Immunology, Albert Einstein College of Medicine, Yeshiva University, Bronx, NY 10461, U.S.A.*

* Corresponding author: Tel. +44 0121 41858125; E-mail: g.besra@bham.ac.uk

**Supporting Information**

**1. General Experimental**

Infrared spectra were recorded neat on a Perkin Elmer Spectrum 100 fitted with a universal ATR accessory. The intensity of each band is described as s (strong), m (medium) or w (weak), and with the prefix v (very) and suffix br (broad) where appropriate. The poor solubility of the amphiphilic α-GalCer analogues **12a-f**, **13**, **14** and **15** at rt prevented us from obtaining reliable optical rotation data. ^1^H NMR spectra were recorded at 500 MHz, 400 MHz or 300 MHz, using Bruker DRX 500, Bruker AMX 400, Bruker AV 400, Bruker AV 300 and Bruker AC 300 spectrometers. ^13^C NMR spectra were recorded at 125 MHz, 100 MHz or 75 MHz, respectively, using Bruker DRX 500, Bruker AMX 400, Bruker AV 400, Bruker AV 300 and Bruker AC 300 spectrometers. Chemical shifts are reported as δ values (ppm) referenced to the following solvent signals: CHCl_3_, δ_H_ 7.26; CDCl_3_, δ_C_ 77.0; CH_3_OH, δ_H_ 3.34; CD_3_OD, δ_C_ 49.0. The term ‘stack’ is used to describe a region where resonances arising from non-equivalent nuclei are coincident, and multiplet, m, to describe a region where a resonance arises from a single nucleus (or equivalent nuclei) but where coupling constants cannot be readily assigned. Mass spectra were recorded on a Micromass LCT spectrometer utilising electrospray ionisation (and a MeOH mobile phase), and are reported as (*m/z* (%)). HRMS were recorded on a Micromass LCT spectrometer using a lock mass incorporated into the mobile phase. All reagents were obtained from commercial sources, and were used without further purification unless stated otherwise. Anhydrous solvents were purchased from Sigma-Aldrich, UK, stored over 4 Å molecular sieves and under an Ar atmosphere. All solutions are aqueous and saturated unless stated otherwise. Reactions were monitored by TLC using pre-coated aluminium-backed ICN silica plates (60A F254) and visualised by UV detection (at 254 nm) and staining with 5% phosphomolybdic acid in EtOH (MPA spray). Column chromatography was performed on Merck silica gel (particle size 40–63 μm mesh) or Fluka 60 (40–60 μm mesh) silica gel.

**2. Experimental Procedures**

**2.1 General procedure for copper-catalysed [3+2] cycloadditions (synthesis of 1,2,3-triazoles 12a-f).** CuSO_4_ solution (5 μL of 0.5 M solution, 2.5 μmol) and sodium ascorbate solution (18 μL of a 1.0 M solution, 18 μmol) were added to a solution of azide **11** (20 mg, 0.023 mmol) and the appropriate alkyne (0.023 mmol) in *^t^*BuOH/H_2_O (1 mL, 1:1) at rt. The reaction mixture was heated for 10 h at 50 °C and then diluted with CHCl_3_ (10 mL), and washed with brine (3 mL). The phases were separated and the aqueous layer was extracted with CHCl_3_ (2 × 5 mL). The combined organic layers were dried over MgSO_4_, filtered and concentrated under reduced pressure. Purification of the residue by flash column chromatography afforded triazoles **12a**-**f**.

**2.2 General procedure for ruthenium-catalysed [3+2] cycloadditions (synthesis of triazoles 13 and 14).** Cp*Ru(PPh_3_)_2_Cl (1.0 mg, 12 μmol) was added to a solution of azide **11** (20 mg, 0.023 mmol) and the appropriate alkyne (0.023 mmol) in degassed THF (1 mL). The resulting mixture was stirred for 3 h at 65 ºC. After removal of the solvent under reduced pressure, purification of the residue by flash column chromatography afforded triazoles **13** and **14**.

**2.3 Preparation of benzotriazole 15.** TBAF (46 μL of a 1 M solution in THF, 0.046 mmol) was added to a solution of azide **11** (20 mg, 0.023 mmol) and 2-(trimethylsilyl)phenyl trifluoromethanesulfonate (11 μL, 0.046 mmol) in THF (1 mL). After stirring the resulting mixture overnight at rt, removal of the solvent under reduced pressure and purification of the residue by flash column chromatography afforded benzotriazole **15** as a white solid.

**3. Characterisation**

**3.1 Triazole 12a.** Azide **11** (20 mg, 0.023 mmol) and phenylacetylene (2.3 mg, 0.023 mmol) were reacted according to General Procedure 2.1. After 10 h, work-up and purification of the residue by flash column chromatography (15% MeOH in CHCl_3_) afforded 1,2,3-triazole **12a** as a colourless paste (21 mg, 94%): *R*_f_ = 0.28 (15% MeOH in CHCl_3_); IR (neat) ν 3371s br (O−H), 2918s, 2850s, 2461w, 1713w, 1629s (C=O), 1557w, 1467s, 1356w, 1230m, 1076s, 1046s, 763m; ^1^H NMR (400 MHz, CDCl_3_:CD_3_OD, 2:1): δ 0.84 (t, 6H, *J* 6.8 Hz, 18-*H*, 26’-*H*), 1.14−1.34 (stack, 68H), 1.42−1.64 (stack, 4H, 5-*H*, 3’-*H*), 2.05 (t, 2H, *J* 7.8 Hz, 2’-*H*), 3.34 (dd, 1H, *J* 10.4, 4.4 Hz, 1-*H*_a_), 3.38−3.45 (stack, 2H, 3-*H*, 4-*H*), 3.52 (dd, 1H, *J* 10.8, 6.0 Hz, 1-*H*_b_), 3.70 (dd, 1H, *J* 10.0, 3.6 Hz, 3”-*H*), 3.79 (dd, 1H, *J* 9.6, 3.6 Hz, 2”-*H*), 3.81−3.85 (m, 1H, 4”-*H*), 4.11 (dt, 1H, *J* 5.2, 4.4 Hz, 2-*H*), 4.23 (dd, 1H, *J* 8.0, 5.2 Hz, 5”-*H*), 4.55−4.67 (stack, 2H, 6”-*H*_a_, 6”-*H*_b_), 4.88 (d, 1H, *J* 3.6 Hz, 1”-*H*), 7.30 (t, 1H, *J* 7.2 Hz, Ph*H*), 7.37−7.43 (m, 2H, Ph*H*), 7.75 (d, 2H, *J* 7.2, Ph*H*), 8.03 (s, 1H, triazole-*H*), exchangeable protons not observed; ^13^C NMR (100 MHz, CDCl_3_:CD_3_OD, 2:1): δ  14.2 (CH_3_, *C*-18, *C*-26’), [22.9, 26.1, 29.6, 30.0, 32.2, 33.1, (CH_2_, alkyl chain, some overlapping resonances)], 36.7 (CH_2_, *C*-2’), 50.2 (CH, *C*-2), 51.2 (CH_2_, C-6”), 67.1 (CH_2_, *C*-1), 68.9 (CH, *C*-2”), 69.6 (CH, *C*-4”), 69.7 (CH, *C*-5”), 70.2 (CH, *C*-3”), 72.1 (CH, *C*-3 or *C*-4), 75.1 (CH, *C*-3 or *C*-4), 99.8 (CH, *C*-1’), 122.1 (CH, triazole), 126.0 (CH, Ph), 128.7 (CH, Ph), 129.2 (CH, Ph), 130.4 (quat. C, Ph), 148.3 (quat. C, triazole), 174.6 (quat. C, *C*-1’); *m/z* (TOF ES+) 1007.6 ([M+Na]^+^, 100%); HRMS *m/z* (TOF ES+) 1007.7764. C_58_H_104_N_4_NaO_8_ requires 1007.7752.

**3.2 Triazole 12b.** Azide **11** (20 mg, 0.023 mmol) and 1-decyne (3.3 mg, 0.023 mmol) were reacted according to General Procedure 2.1. After 10 h, work-up and purification of the residue by flash column chromatography (15% MeOH in CHCl_3_) afforded 1,2,3-triazole **12b** as a colourless paste (22 mg, 95%): *R*_f_ = 0.30 (15% MeOH in CHCl_3_); IR (neat) ν 3385m br (O–H), 2916s, 2852s, 1635m (C=O), 1532w, 1468s, 1438w, 1345w, 1204m, 1151s, 1065s, 1036s, 801m, 732m, 725m; ^1^H NMR (300 MHz, CDCl_3_:CD_3_OD, 2:1): δ 0.84 (app. t, 9H, *J* 6.6 Hz), 1.12−1.43 (stack, 78H), 1.47−1.71 (stack, 6H), 2.13 (app. t, 2H, *J* 7.7 Hz), 2.64 (app. t, 2H, *J* 7.8 Hz), 3.37 (dd, 1H, *J* 10.5, 4.5 Hz), 3.42−3.55 (stack, 3H), 3.68 (dd, 1H, *J* 10.2, 3.0 Hz), 3.75−3.84 (stack, 2H), 4.07− (stack, 2H), 4.51−4.57 (m, 2H), 4.86 (d, 1H, *J* 3.6 Hz), 7.29 (d, 1H, *J* 8.7 Hz), 7.52 (s, 1H), alcoholic protons not observed; ^13^C NMR (75 MHz, CDCl_3_:CD_3_OD, 2:1): δ 14.3 (CH_3_), [23.0, 25.8, 26.3, 29.7, 29.8, 30.1, 32.3, 33.0, 36.8 (CH_2_, some overlapping alkyl resonances)], 50.4 (CH), 51.1 (CH_2_), 67.4 (CH_2_), 69.1 (CH), 69.8 (CH), 69.9 (CH), 70.3 (CH), 72.3 (CH), 75.1 (CH), 100.0 (CH), 123.17 (CH), 148.6 (quat. C), 174.7 (quat. C); *m/z* (TOF ES+) 1043.9 ([M+Na]^+^, 100%); HRMS *m/z* (TOF ES+) 1043.8697. C_60_H_116_N_4_NaO_8_ requires 1043.8691.

**3.3 Triazole 12c.** Azide **11** (20 mg, 0.023 mmol) and 1-tetradecyne (4.5 mg, 0.023 mmol) were reacted according to General Procedure 2.1. After 10 h, work-up and purification of the residue by flash column chromatography (15% MeOH in CHCl_3_) afforded 1,2,3-triazole **12c** as a colourless paste (23 mg, 92%): *R*_f_ = 0.31 (15% MeOH in CHCl_3_); IR (neat) ν 3387m br (O–H), 2917s, 2853s, 1635m (C=O), 1530w, 1468s, 1438w, 1352w, 1345w, 1204m, 1151s, 1065s, 1061m, 1036s, 802m, 732m, 725m; ^1^H NMR (400 MHz, CDCl_3_:CD_3_OD, 2:1): δ 0.84 (app. t, 9H, *J* 6.8 Hz), 1.15−1.42 (stack, 86H), 1.48−1.69 (stack, 6H), 2.12 (app. t, 2H, *J* 7.6), 2.64 (dd, 2H, *J* 8.0, 7.6 Hz), 3.37 (dd, 1H, *J* 10.8, 4.4 Hz), 3.43−3.47 (stack, 2H), 3.51 (dd, 1H, *J* 10.4, 4.5 Hz), 3.68 (dd, 1H, *J* 10.1, 3.1 Hz), 3.75−3.79 (stack, 2H), 4.08−4.13 (m, 1H), 4.16 (dd, 1H, *J* 11.6, 5.2 Hz), 4.47 (dd, 1H, *J* 14.0, 8.0 Hz), 4.54 (dd, 1H, *J* 14.0, 4.8 Hz), 4.85 (d, 1H, *J* 4.0), 7.50 (s, 1H), exchangeable protons not observed; ^13^C NMR (75 MHz, CDCl_3_:CD_3_OD, 2:1): δ 14.4 (CH_3_), [23.1, 25.9, 26.4, 29.8, 29.9, 30.2, 32.4, 33.1, 36.9 (CH_2_, some overlapping alkyl resonances)], 50.5 (CH), 51.2 (CH_2_), 67.5 (CH_2_), 69.1 (CH), 69.9 (CH), 70.0 (CH), 70.4 (CH), 72.3 (CH), 75.2 (CH), 100.1 (CH), 123.3 (CH), 148.7 (quat.C), 174.8 (quat. C); *m/z* (TOF ES+) 1099.9 ([M+Na]^+^, 100%); HRMS *m/z* (TOF ES+) 1099.9306. C_64_H_124_N_4_NaO_8_ requires 1099.9317.

**3.4 Triazole 12d.** Azide **11** (20 mg, 0.023 mmol) and pent-1-yn-3-one (1.9 mg, 0.023 mmol) were reacted according to General Procedure 2.1. After 10 h, work-up and purification of the residue by flash column chromatography (15% MeOH in CHCl_3_) afforded 1,2,3-triazole **12d** as a colourless paste (20 mg, 89%): *R*_f_ = 0.28 (15% MeOH in CHCl_3_); IR (neat) ν  3372s br (O−H), 2916s, 2851s, 2459w, 1631s (C=O), 1554w, 1550w, 1467s, 1356w, 1230m, 1075s, 1071w, 1046s, 763m, 760m; ^1^H NMR (400 MHz, CDCl_3_:CD_3_OD, 2:1): δ 0.84 (t, 6H, *J* 6.8 Hz), 1.17 (t, 3H, *J* 7.3 Hz), 1.17-1.32 (stack, 68H), 1.42-1.67 (stack, 4H), 2.10-2.17 (m, 2H), 3.08 (q, 2H, *J* 7.3 Hz), 3.36 (dd, 1H, *J* 10.0, 3.6 Hz), 3.41-3.51 (stack, 3H), 3.70 (dd, 1H, *J* 10.0, 3.2 Hz), 3.78 (dd, 1H, *J* 10.0, 3.6, Hz), 3.80-3.83 (m, 1H), 4.09 (app. q, 1H, *J* 4.8 Hz), 4.18-4.22 (m, 1H), 4.59-4.63 (stack, 2H), 4.85 (d, 1H, *J* 3.6 Hz), 8.03 (s, 1H), alcoholic protons not observed; ^13^C NMR (100 MHz, CDCl_3_:CD_3_OD, 2:1): δ 7.8 (CH_3_), 14.2 (2 × CH_3_), [23.0, 26.2, 29.7, 30.0, 32.3, 33.0, 33.2, 36.8 (CH_2_, some overlapping alkyl resonances)], 50.3 (CH), 51.5 (CH_2_), 67.6 (CH_2_), 68.9 (CH), 69.6 (CH), 69.7 (CH), 70.2 (CH), 72.3 (CH), 75.0 (CH), 100.0 (CH), 127.7 (CH), 147.5 (quat. C), 174.7 (quat. C), 196.4 (quat. C); *m/z* (TOF ES+) 987.8 ([M+Na]^+^, 100%); HRMS *m/z* (TOF ES+) 987.7705. C_55_H_104_N_4_NaO_9_ requires 987.7701.

**3.5 Triazole 12e.** Azide **11** (20 mg, 0.023 mmol) and methyl propargyl ether (1.6 mg, 0.023 mmol) were reacted according to General Procedure 2.1. After 10 h, work-up and purification of the residue by flash column chromatography (15% MeOH in CHCl_3_) afforded 1,2,3-triazole **12e** as a colourless paste (20 mg, 92%): *R*_f_ = 0.29 (15% MeOH in CHCl_3_); IR (neat) ν 3352br s (O–H), 2914s, 2851s, 1638m (C=O), 1543w, 1467m, 1343w, 1229w, 1150m, 1067s, 1034s, 784w, 720s, 717w, 668m; ^1^H NMR (300 MHz, CDCl_3_:CD_3_OD, 2:1): δ 0.84 (app. t, 6H, *J* 6.6 Hz), 1.14−1.38 (stack, 68H), 1.43−1.67 (stack, 4H), 2.13 (app. t, 2H, *J* 7.5 Hz), 3.33−3.40 (stack, [including 3.37 (s, 3H)], 4H), 3.42−3.52 (stack, 3H), 3.69 (dd, 1H, *J* 9.8, 3.3 Hz), 3.74−3.83 (stack, 2H), 4.03−4.13 (m, 1H), 4.13−4.20 (m, 1H), 4.52 (s, 2H), 4.53−4.59 (m, 2H), 4.85 (d, 1H, *J* 3.7 Hz), 7.28 (d, 1H, *J* 8.8 Hz), 7.78 (s, 1H), alcoholic protons not observed; ^13^C NMR (75 MHz, CDCl_3_:CD_3_OD, 2:1): δ 14.2 (CH_3_), [23.0, 26.2, 29.7, 30.0, 32.3, 33.0, 36.8 (CH_2_, overlapping alkyl resonances)], 50.5 (CH), 51.3 (CH_2_), 58.4 (CH_3_), 65.8 (CH_2_), 67.5 (CH_2_), 69.0 (CH), 69.7 (CH), 69.8 (CH), 70.3 (CH), 72.3 (CH), 75.1 (CH), 100.0 (CH), 125.0 (CH), 144.8 (quat. C), 174.7 (quat. C); *m/z* (TOF ES+) 975.8 ([M+Na]^+^, 100%); HRMS *m/z* (TOF ES+) 975.7711. C_54_H_104_N_4_NaO_9_ requires 975.7701.

**3.6 Triazole 12f.** Azide **11** (20 mg, 0.023 mmol) and *O*-methyl-*O*’-propargyl-octa(ethylene glycol) (9.7 mg, 0.023 mmol) were reacted according to General Procedure 2.1. After 10 h, work-up and purification of the residue by flash column chromatography (20% MeOH in CHCl_3_) afforded 1,2,3-triazole **12f** as a colourless paste (29 mg, 97%): *R*_f_ = 0.27 (20% MeOH in CHCl_3_); IR (neat) ν 3336m br (O–H), 2919s, 2849s, 1630m (C=O), 1549w, 1467m, 1350m, 1301w, 1231w, 1138m, 1082s, 1039s, 1012w, 948m, 786m, 719s; ^1^H NMR (300 MHz, CDCl_3_:CD_3_OD, 2:1): δ 0.84 (app. t, 6H, *J* 6.6 Hz), 1.12−1.39 (stack, 68H), 1.45−1.69 (stack, 4H), 2.13 (app. t, 2H, *J* 7.4 Hz), 3.33−3.41 (stack, [including 3.35 (s, 3H)], 4H), 3.41−3.50 (stack, 3H), 3.50−3.56 (stack, 2H), 3.58−3.65 (stack, 30H), 3.69 (dd, 1H, *J* 10.2, 3.0 Hz), 3.74−3.81 (stack, 2H), 4.05−4.13 (m, 1H), 4.18 (app. t, 1H, *J* 6.6 Hz), 4.52−4.58 (m, 2H), 4.62 (s, 2H), 4.85 (d, 1H, *J* 3.6 Hz), 7.28 (d, 1H, *J* 8.7 Hz), 7.80 (s, 1H), alcoholic protons not observed; ^13^C NMR (75 MHz, CDCl_3_:CD_3_OD, 2:1): δ 14.2 (CH_3_), [23.0, 26.2, 29.7, 30.0, 32.3, 33.0, 36.8 (CH_2_, some overlapping alkyl resonances)], 50.4 (CH), 51.2 (CH_2_), 59.1 (CH_3_), 64.5 (CH_2_), 67.5 (CH_2_), 69.0 (CH), 69.7 (CH), 69.8 (CH), 70.0 (CH_2_), 70.3 (CH), 70.8 (CH_2_, some overlapping ethylene glycol resonances), 72.2 (CH_2_), 72.3 (CH), 75.0 (CH), 100.0 (CH), 125.1 (CH), 145.0 (quat. C), 174.7 (quat. C); *m/z* (TOF ES+) 1328.8 ([M+Na]^+^, 100%); HRMS *m/z* (TOF ES+) 1327.9788. C_70_H_136_N_4_NaO_17_ requires 1327.9798.

**3.7 Triazole 13.** Azide **11** (20 mg, 0.023 mmol) and phenylacetylene (2.3 mg, 0.023 mmol) were reacted according to General Procedure 2.2. After 3 h, concentration of the reaction mixture and purification of the residue by flash column chromatography (15% MeOH in CHCl_3_) afforded 1,2,3-triazole **13** as a colourless paste (18 mg, 78%): *R*_f_ = 0.28 (15% MeOH in CHCl_3_); IR (neat) ν  3373s br (O−H), 2916s, 2849s, 2460w, 1713w, 1631s (C=O), 1554w, 1466s, 1359w, 1246m, 1238w, 1230m, 1076s, 1046s, 763m, 724m, 713m; ^1^H NMR (400 MHz, CDCl_3_:CD_3_OD, 2:1): δ 0.84 (app. t, 6H, *J* 6.9 Hz, 18-*H*, 26’-*H*), 1.17−1.33 (stack, 68H, alkyl chain), 1.43−1.68 (stack, 4H, 5-*H*, 3’-*H*), 2.15 (dd, 2H, *J* 10.8, 7.6 Hz, 2’-*H*), 3.26−3.34 (m, 1H, 1-*H*_a_), 3.43−3.54 (stack, 3H, 1-*H*_b_, 3-*H*, 4-*H*), 3.71 (dd, 1H, *J* 10.0, 2.8 Hz, 3”-*H*), 3.74 (dd, 1H, *J* 10.0, 3.6 Hz, 2”-*H*), 3.84−3.89 (m, 1H, 4”-*H*), 4.13 (dd, 1H, *J* 9.6, 4.8 Hz, 2-*H*), 4.42−4.50 (stack, 2H, 5”-*H*, 6”-*H*_a_), 4.58 (dd, 1H, *J* 14.8, 9.6 Hz, 6”-*H*_b_), 4.78 (d, 1H, *J* 3.2 Hz, 1”-*H*), 7.43−7.49 (stack, 5H, Ph*H*), 7.65 (s, 1H, triazole-*H*), exchangeable protons not observed; ^13^C NMR (75 MHz, CDCl_3_:CD_3_OD, 2:1): δ 14.3 (CH_3_, *C*-18, *C*-26”), [23.0, 26.3, 29.7, 29.8, 30.1, 32.3, 33.0 (CH_2_, alkyl chain, some overlapping resonances)], 36.8 (CH_2_, *C*-2’), 49.1 (CH_2_, *C*-6”), 50.4 (CH, C-2), 67.3 (CH_2_, *C*-1), 69.0 (CH, *C*-3”), 69.8 (CH, C-4”), 70.0 (CH, *C*-5”), 70.4 (CH, *C*-2”), 72.3 (CH, *C*-3 or *C*-4), 75.2 (CH, *C*-3 or *C*-4), 99.9 (CH, *C*-1”), 126.8 (quat. C, Ph), 129.5 (2 × CH, overlapping resonances, Ph), 130.1 (CH, Ph), 132.9 (CH, triazole), 140.0 (quat. C, triazole), 174.8 (quat. C, *C*-1’); *m/z* (TOF ES+) 1007.6 ([M+Na]^+^, 100%); HRMS *m/z* (TOF ES+) 1007.7746. C_58_H_104_N_4_NaO_8_ requires 1007.7752.

**3.8 Triazole 14.** Azide **11** (20 mg, 0.023 mmol) and diphenylacetylene (4.1 mg, 0.023 mmol) were reacted according to General Procedure 2.2. After 3 h, concentration of the reaction mixture and purification of the residue by flash column chromatography (15% MeOH in CHCl_3_) afforded 1,2,3-triazole **14** as a colourless paste (18 mg, 72%): *R*_f_ = 0.28 (15% MeOH in CHCl_3_); IR (neat) ν  3370s br (O−H), 2920s, 2851s, 2460w, 1711w, 1630s (C=O), 1557w, 1467s, 1356w, 1242w, 1230m, 1092w, 1084w, 1076s, 1053m, 1046s, 767m, 757m; ^1^H NMR (300 MHz, CDCl_3_:CD_3_OD, 2:1): δ 0.84 (app. t, 6H, *J* 7.2), 1.13−1.34 (stack, 68H), 1.42−1.69 (stack, 4H), 2.11 (app. t, 2H, *J* 7.7 Hz), 3.45−3.53 (stack, 2H), 3.57 (dd, 1H, *J* 10.5, 5.7 Hz), 3.58−3.77 (stack, 3H), 3.85−3.89 (m, 1H), 4.09−4.21 (stack, 2H), 4.36−4.41 (stack, 2H), 4.81 (d, 1H, *J* 2.4 Hz), 7.19−7.28 (m, 2H), 7.30−7.38 (m, 2H), 7.39−7.45 (m, 2H), 7.45−7.52 (stack, 4H), exchangeable protons not observed; ^13^C NMR (75 MHz, CDCl_3_:CD_3_OD, 2:1): δ 14.6 (CH_3_), [23.3, 26.1, 26.6, 30.0, 30.1, 30.4, 32.6, 33.4, 37.1 (CH_2_, some overlapping alkyl resonances)], 48.9 (CH_2_), 50.7 (CH), 67.7 (CH_2_), 69.4 (CH), 70.1 (CH), 70.2 (CH), 70.6 (CH), 72.6 (CH), 75.4 (CH), 100.3 (CH), 126.8 (CH), 127.6 (CH), 128.4 (CH), 129.4 (CH), 130.18 (CH), 130.22 (CH), 131.2 (quat. C), 132.5 (quat. C), 144.0 (quat. C), 175.0 (quat. C), one quaternary carbon not observed due to resonance overlap; *m/z* (TOF ES+) 1083.8 ([M+Na]^+^, 100%); HRMS *m/z* (TOF ES+) 1083.8062. C_64_H_108_N_4_NaO_8_ requires 1083.8065.

**3.9 Benzotriazole 15.** TBAF (46 μL of a 1 M solution in THF, 0.046 mmol), azide **11** (20 mg, 0.023 mmol) and 2-(trimethylsilyl)phenyl trifluoromethanesulfonate (11 μL, 0.046 mmol) were reacted as described in Procedure 2.3. After overnight stirring, removal of the solvent under reduced pressure and purification of the residue by flash column chromatography afforded benzotriazole **15** as a white solid (15 mg, 69%): *R*_f_ = 0.29 (15% MeOH in CHCl_3_); IR (film) ν 3369s br (O−H), 2918s, 2851s, 2461w, 1714w, 1628s (C=O), 1552w, 1470s, 1360w, 1254w, 1229m, 1086m, 1077w, 1073s 1054w, 1046s, 997w, 763m, 755m, 743s; ^1^H NMR (500 MHz, CDCl_3_:CD_3_OD, 2:1): δ 0.83 (t, 6H, *J* 7.0 Hz), 1.15-1.35 (stack, 68H), 1.41-1.63 (stack, 4H), 2.05−2.11 (stack, 2H), 3.18 (dd, 1H, *J* 11.0, 4.5 Hz), 3.25 (dd, 1H, *J* 9.0, 4.0 Hz), 3.36−3.43 (stack, 2H), 3.68 (dd, 1H, *J* 10.0, 3.5 Hz), 3.79 (dd, 1H, *J* 10.0, 4.0 Hz), 3.81−3.83 (m, 1H), 3.91-3.93 (m, 1H), 4.01 (app. q, 1H, *J* 4.0 Hz), 4.80 (d, 1H, *J* 3.9 Hz), 4.81−4.87 (stack, 2H), 7.33−7.38 (m, 1H), 7.46−7.51 (m, 1H), 7.71 (d, 1H, *J* 8.5 Hz), 7.93 (d, 1H, *J* 8.5 Hz), exchangeable protons not observed; ^13^C NMR (100 MHz, CDCl_3_:CD_3_OD, 2:1): δ 15.2 (CH_3_), [24.0, 27.1, 30.7, 31.0, 33.2, 33.9, 35.1, 37.7 (CH_2_, some overlapping alkyl resonances)], 50.1 (CH_2_), 51.2 (CH), 68.2 (CH_2_), 70.0 (CH), 70.8 (CH), 71.2 (CH), 71.3 (CH), 73.2 (CH), 75.9 (CH), 100.8 (CH), 111.9 (CH), 120.3 (CH), 125.8 (CH), 129.1 (CH), 138.4 (quat. C), 155.6 (quat. C), 175.5 (quat. C); *m/z* (TOF ES+) 981.6 ([M+Na]^+^, 100%); HRMS *m/z* (TOF ES+) 981.7616. C_56_H_102_N_4_NaO_8_ requires 981.7595.

**4. Biological Methods**

For *in vitro* stimulation, murine *i*NKT hybridoma DN3A4-1.2 at 2.5 x 10^4^ cells/well in 96 well plates were stimulated with an equal number of murine bone-marrow derived dendritic cells (C57BL/6 strain) in complete medium (RPMI-1640 with 1 mM HEPES, 50 μM 2-mercaptoethanol and 10% fetal bovine serum) with glycolipids for 24 h at 37 °C, and levels of murine IL-2 secretion were determined using standard capture ELISA.^1^ *In vivo* activation of *i*NKT cells by i.p. injection of glycolipids (4 nmoles) of female C57BL/6 mice and measurement of serum cytokines (IFNγ and IL-4) by capture ELISA was performed as previously described.^2^

**References**

1. Im, J. S.; Arora, P.; Bricard, G.; Molano, A.; Venkataswamy, M. M.; Baine, I.; Jerud, E. S.; Goldberg, M. F.; Yu, K.O.A.; Ndonye, R. M.; Howell, A. R.; Yuan, W.; Cresswell, P.; Chang, Y. T.; Illarionov, P. A.; Besra G. S.; Porcelli, S. A. *Immunity* **2009**, *30*, 888−898.
2. Forestier, C.; Takaki, T,; Molano, A.; Im, J.S.; Baine, I.; Jerud, E. S.; Illarionov, P.; Ndonye, R.; Howell, A. R.; Santamaria, P.; Besra, G. S.; DiLorenzo, T. P.; Porcelli, S. A. *J. Immunol.* **2007**, *178*, 1415−1425.
